# A homozygous *POLR1A* variant causes leukodystrophy and affects protein homeostasis

**DOI:** 10.1093/brain/awad086

**Published:** 2023-03-14

**Authors:** Doriana Misceo, Lisa Lirussi, Petter Strømme, Dulika Sumathipala, Andrea Guerin, Nicole I Wolf, Andres Server, Maria Stensland, Bjørn Dalhus, Aslıhan Tolun, Hester Y Kroes, Tuula A Nyman, Hilde L Nilsen, Eirik Frengen

**Affiliations:** Department of Medical Genetics, Oslo University Hospital and University of Oslo, 0450 Oslo, Norway; Department of Clinical Molecular Biology, University of Oslo, 0318 Oslo, Norway; Section of Clinical Molecular Biology (EpiGen), Akershus University Hospital, 1478 Lørenskog, Norway; Division of Pediatric and Adolescent Medicine, Oslo University Hospital and University of Oslo, 0450 Oslo, Norway; Department of Medical Genetics, Oslo University Hospital and University of Oslo, 0450 Oslo, Norway; Kingston Health Sciences Centre, Queen's Medical School, Kingston, ON K7L 2V7, Canada; Department of Child Neurology, Amsterdam Leukodystrophy Center, Emma Children's Hospital, Amsterdam University Medical Centers, Vrije Universiteit Amsterdam, and Amsterdam Neuroscience, 1081 HV Amsterdam, the Netherlands; Section of Neuroradiology, Department of Radiology and Nuclear Medicine, Oslo University Hospital, Rikshospitalet, 0424 Oslo, Norway; Department of Immunology, Oslo University Hospital and Faculty of Medicine, University of Oslo, 0372 Oslo, Norway; Department for Microbiology, Oslo University Hospital, 0424 Oslo, Norway; Department for Medical Biochemistry, University of Oslo, 0424 Oslo, Norway; Department of Molecular Biology and Genetics, MOBGAM, Istanbul Technical University, 34469 Istanbul, Turkey; Department of Genetics, UMC, 3584 CX Utrecht, the Netherlands; Department of Immunology, Oslo University Hospital and Faculty of Medicine, University of Oslo, 0372 Oslo, Norway; Department of Clinical Molecular Biology, University of Oslo, 0318 Oslo, Norway; Section of Clinical Molecular Biology (EpiGen), Akershus University Hospital, 1478 Lørenskog, Norway; Department for Microbiology, Oslo University Hospital, 0424 Oslo, Norway; Department of Medical Genetics, Oslo University Hospital and University of Oslo, 0450 Oslo, Norway

**Keywords:** POLR1A, rRNA, ribosome, myelin, neurodegeneration

## Abstract

RNA polymerase I transcribes ribosomal DNA to produce precursor 47S rRNA. Post-transcriptional processing of this rRNA generates mature 28S, 18S and 5.8S rRNAs, which form the ribosomes, together with 5S rRNA, assembly factors and ribosomal proteins. We previously reported a homozygous variant in the catalytic subunit of RNA polymerase I, POLR1A, in two brothers with leukodystrophy and progressive course. However, the disease mechanism remained unknown.

In this report, we describe another missense variant *POLR1A* NM_015425.3:c.1925C>A; p.(Thr642Asn) in homozygosity in two unrelated patients. Patient 1 was a 16-year-old male and Patient 2 was a 2-year-old female. Both patients manifested neurological deficits, with brain MRIs showing hypomyelinating leukodystrophy and cerebellar atrophy; and in Patient 1 additionally with hypointensity of globi pallidi and small volume of the basal ganglia. Patient 1 had progressive disease course, leading to death at the age of 16.5 years. Extensive *in vitro* experiments in fibroblasts from Patient 1 documented that the mutated POLR1A led to aberrant rRNA processing and degradation, and abnormal nucleolar homeostasis. Proteomics data analyses and further *in vitro* experiments documented abnormal protein homeostasis, and endoplasmic reticulum stress responses. We confirm that *POLR1A* biallelic variants cause neurodegenerative disease, expand the knowledge of the clinical phenotype of the disorder, and provide evidence for possible pathological mechanisms leading to POLR1A-related leukodystrophy.

## Teaser

Misceo *et al*. describe two patients with defective *POLR1A*, both presenting with leukodystrophy and cerebellar atrophy. Experiments in patient fibroblasts reveal aberrant rRNA processing, nucleolar stress, and impaired protein homeostasis, providing new information about the molecular basis of *POLR1A*-related leukodystrophy.

## Introduction

The eukaryotic genome is transcribed by three large multi-subunit complexes known as RNA polymerases I, II and III, responsible for the transcription of different classes of RNAs.^1^ In the nucleolus, RNA polymerase I transcribes ribosomal DNA (rDNA) loci to produce the precursor 47S rRNA. This molecule undergoes a series of endo- and exonucleolytic processing steps and chemical modifications to form mature rRNAs 28S, 18S and 5.8S. These rRNAs, together with 5S rRNA, assembly factors and ribosomal proteins, form the ribosomes dedicated to protein synthesis. RNA polymerase I activity is the limiting factor for ribosome biogenesis.^2^ RNA polymerase II is responsible for the transcription of all protein-encoding genes and some non-coding RNA genes, whereas RNA polymerase III synthetizes small non-coding RNAs including tRNAs, 5S rRNA and microRNAs.^1^

Deleterious variants in several genes that encode subunits of the RNA polymerase I or RNA polymerase III complexes cause Mendelian diseases, falling mainly in two groups: leukodystrophies and skeletal diseases. In particular, heterozygous variants in the catalytic unit of RNA polymerase I, POLR1A, cause acrofacial dysostosis, Cincinnati type (MIM 616462), described in four unrelated patients exhibiting varying mandibulofacial dysostosis phenotypes, with or without extra-facial skeletal defects.^3,4^ As for the recessive disease, we previously reported two brothers, homozygous for *POLR1A* NM_015425.3:c.2801C>T, p.(Ser934Leu), with leukodystrophy and atrophy of cerebrum, cerebellum and corpus callosum, leading to spasticity and in the elder brother additionally neurological regression.^5^

We now report two unrelated patients, both homozygous for missense variant POLR1A NM_015425.3:c.1925C>A; p.(Thr642Asn) who presented at an early age with global developmental delay, accompanied by hypomyelinating leukodystrophy and cerebellar degeneration. In the older patient, the neurological manifestations were progressive, from the age of 5 years, with fatal outcome at 16.5 years. We performed *in silico* and *in vitro* studies, documenting abnormal rRNA processing and degradation, and nucleolar stress, probably causing the observed abnormal protein homeostasis and endoplasmic reticulum (ER) stress responses. In conclusion, we confirm that biallelic pathogenic *POLR1A* variants cause leukodystrophy with recessive inheritance, and provide for the first-time preliminary insight into possible cellular consequences of POLR1A dysfunction.

## Materials and methods

### Ethical considerations

For Patient 1 the study was approved by the Regional Committee for Medical Research Ethics—South-East Norway, REK 2010/1152a. The genetic study in Patient 2 was part of a diagnostic workup and thus did not require ethical approval. Written informed consent was obtained from the parents of both patients to perform genetic testing and to publish clinical information.

### Genetic studies

Exome sequencing was performed to detect candidate variants and Sanger sequencing to validate the variant.

For Patient 1, genomic DNA was extracted from cultured skin fibroblasts of patient and from blood in parents and sister. Exome sequencing of Patient 1 and the parents was performed on Illumina HiSeq2000 instrument (Illumina Inc.) with 100 bp paired-end reads. Exome sequencing and data analysis were performed as previously described.^6^ Sanger sequencing was done on the PCR product generated with primer pair forward 5′-TGTGGGCAAGACAAGACAGGGA-3′ and reverse 5′-ACTCCCTTGAAGCCACCCAGAC-3′, using ABI BigDye dye terminator cycle-sequencing kits v3.1 and ABI 3730xl DNA analyser (Life Technologies).

For Patient 2, after referral for routine diagnostic exome sequencing, exomes from the child and parents were enriched using the Agilent SureSelect XT Human All Exon kit CREv2 (Agilent Technologies) and sequenced at 2 × 150 bp on a NovaSeq 6000 sequencing system (Illumina Inc.) at a mean target depth of 160×. Reads were aligned and variants were called using an in house developed pipeline (IAP v2.6.1, github.com/UMCUGenetics/IAP/tree/v2.6.1). Detected variants were annotated, filtered and prioritized using the Bench NGS Laboratory platform (Agilent Technologies). Exome sequencing for Patient 2 and healthy parents was performed in the Genome Diagnostics laboratory of the University Medical Center, Utrecht.

### Structural modelling

The Alpha Fold-predicted 3D structure^7^ of human RNA polymerase I subunits RPA1 and RPABC3 were retrieved from the UniProt Database entries O95602 and P52434, respectively, and superimposed onto the corresponding subunits in the 3.4 Å resolution cryogenic-electron microscopy structure of the bovine RNA polymerase II complex (1FLM.pdb)^8^ using the PyMol molecular graphics system (Schrödinger LLC). The position of POLR1A variant T642N was inspected using this model of the RNA polymerase I complex.

### Cell cultures and treatments

Fibroblasts were obtained from skin biopsy taken from Patient 1 at the age of 16 years, and from two controls at 37 and 42 years, female and male, respectively. Fibroblasts were maintained in culture in Dulbecco's modified Eagle medium (Life Technologies) supplemented with 10% (vol/vol) foetal bovine serum, 100 U/ml penicillin and 100 μg/ml streptomycin sulphate. Cells were cultured at 37°C under 5% CO_2_.

Etoposide (Etop), Bafilomycin A1 (BFA), cycloheximide (CHX) and thapsigargin were purchased from Sigma-Aldrich and suspended in dimethyl sulphoxide.

For ionizing radiation treatment, cells were seeded onto coverslips in a 24-well cell culture dish and irradiated with 2 Gy. After 6 h, immunofluorescence experiments were conducted.

### Immunofluorescence and confocal microscopy

Immunofluorescence experiments were carried out as described previously,^9^ with minor modifications. Nucleolar size was assessed and quantified by measuring NPM1 immunostaining with Squassh software.^10^ The list of antibodies used is provided in Supplementary Table 1.

### Western assays

Western assays on whole-cell lysates were performed as described previously,^9^ with minor modifications. Blots were developed with SuperSignal West Pico or Femto Chemiluminescent substrates (Thermo Scientific). Signals were detected with a ChemiDoc imaging system (Bio-Rad) and quantified with ImageJ software. The list of antibodies used is available in Supplementary Table 1.

### Assessment of nucleolar transcription

Fluorine-conjugated UTP (FUrd) incorporation was performed as described previously.^11,12^

### Chromatin immunoprecipitation

Chromatin immunoprecipitation (ChIP) was performed essentially as described in Dahl and Collas (2008).^13^ Purified DNA was analysed via quantitative polymerase chain reaction (qPCR). Fold enrichment as percentage of input was calculated by normalizing ChIP reactions to input DNA of the target gene. Primers used for ChIP experiment were described.^14^

### Telomere length analysis

Average telomere length was measured from total genomic DNA samples by using the qPCR method,^15^ with modifications as described previously.^9^

### RNA isolation, cDNA synthesis and quantitative PCR

Total RNA was isolated with RNeasy kit (Qiagen) following the manufacturer's instructions. Reverse transcription was performed using iScript™ cDNA Synthesis kit (Bio-Rad). Quantitative PCR was carried out on a QuantStudio 7 Flex detection system (Applied Biosystems) with Power SYBR green PCR master mix (Applied Biosystems). Each sample was analysed in triplicate. Primers used are available on request.

### CpG methylation analysis

Methyl CpG content at −59 of rDNA promoter sequence was measured as described previously.^15^ Briefly, genomic DNA was digested overnight at 25°C with SmaI, and after heat inactivation, DNA was purified using phenol-chloroform-isoamyl alcohol mixture. The CpG methylation levels were then quantified by qPCR using the primers described previously.^15^

### Statistical analysis for *in vitro* studies

All quantified data are presented as mean ± SEM, mean ± SD or fold change unless stated otherwise. To assess statistical significance, Student’s *t*-test or one-way ANOVA was used in Microsoft Excel or GraphPad Prism 7 (GraphPad software). A *P-*value < 0.05 was considered to be statistically significant.

### Label-free quantitative proteomics and data analysis

Cell nuclear and cytoplasmic extracts were prepared as previously described.^16^ Proteins were precipitated with 2-D Clean Up-Kit (GE Healthcare) according to the manufacturer's instructions. The pellets were suspended in 40 µl of 50 mM NH_4_HCO_3_ with 0.2% ProteaseMAX Surfactant (Promega), and protein concentration was measured with Direct Detect (Millipore). Proteins were reduced, alkylated and digested into peptides with trypsin (Promega) according to the ProteaseMAX™ protocol. The resulting peptides were desalted and concentrated before label-free quantitative proteomics by the STAGE-TIP method using a C18 resin disc (3M Empore). Each peptide mixture was analysed by an nEASY-LC coupled to QExactive Plus (ThermoElectron) with EASY Spray PepMap®RSLC column (C18, 2 µl, 100 Å, 75 µm × 50 cm) using a 120-min liquid chromatography (LC) separation gradient. The resulting MS raw files were submitted to the MaxQuant software v.1.5.3.8 for protein identification and label-free quantification. Carbamidomethyl (C) was set as a fixed modification and acetyl (protein N-term), carbamyl (N-term) and oxidation (M) were set as variable modifications. First search peptide tolerance of 20 ppm and main search error 4.5 ppm were used. Trypsin without proline restriction enzyme option was used, with two allowed miscleavages. The minimal unique + razor peptides number was set to 1, and the allowed FDR was 0.01 (1%) for peptide and protein identification. Label-free quantitation was employed with default settings. The Perseus software^17^ (v.1.6.1.3) was used for statistical analysis of the MaxQuant results. First, known contaminants as provided by MaxQuant and identified in the samples were excluded from further analysis. Then, nuclear or cytoplasmic protein levels of Patient 1 versus Control 1 or Control 2 were analysed for statistically significant differential expression (Student's *t*-test with permutation-based FDR <0.05) The proteomics results were further analysed using Ingenuity Pathway Analysis (IPA) (Qiagen).

### Data availability

The label-free quantitative proteomics data have been deposited to the ProteomeXchange Consortium via the PRIDE^17^ partner repository with the dataset identifier PXD017431.

Other data are available on reasonable request. Distribution of sensitive data is subject to restrictions.

## Results

### Clinical data

**Figure 1:**
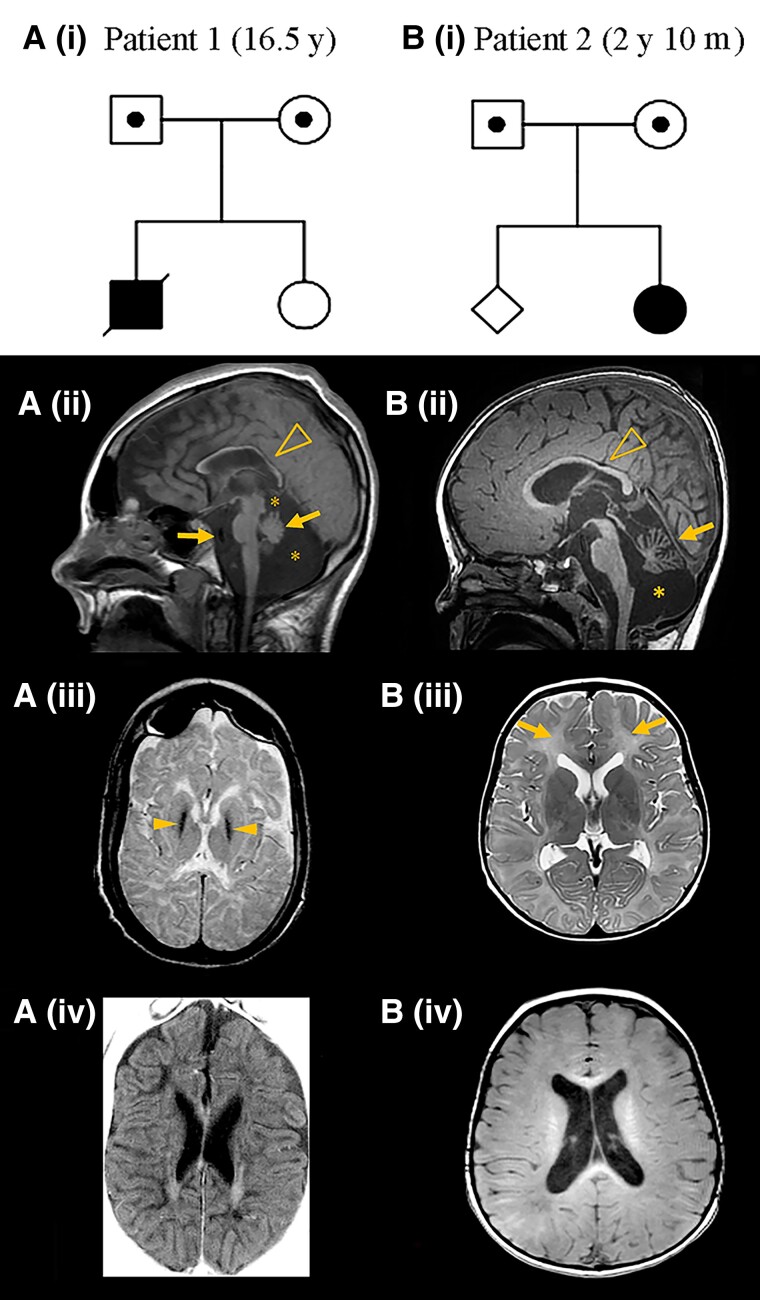
**Family pedigrees, *POLR1A* variant segregation and brain MRI of patients**. [**A**(**i**) and **B**(**i**)] The POLR1A variant status is indicated as homozygous (filled symbols) and heterozygous (dotted symbols) in the pedigrees. Age of the patient at MRI examination is indicated in parenthesis. [**A**(**ii**)] Midline sagittal T_1_-weighted image shows a thin corpus callosum (arrowhead), a small cerebellum (arrow), prominent cisterns (asterisks), mild scalloping of the occipital bone, decreased pontine area, thin medulla oblongata and enlarged retroclival CSF space (arrow). [**A**(**iii**)] Axial T_2_-weighted image shows marked hypointensity of the globi pallidi (arrowheads). Basal ganglia and thalami have a small volume. [**A**(**iv**)] Axial T_1_-weighted image shows enlarged lateral ventricles and subarachnoid spaces with a reduced volume of white matter, as well as hypointensity of the supratentorial white matter, suggesting hypomyelination with some residual T_1_ hyperintensity parallel to the lateral ventricles. [**B**(**ii**)] Midline sagittal T_1_-weighted image shows a mildly thin corpus callosum (arrowhead), a small cerebellum (arrow) and an enlarged cisterna magna (asterisk), with additional evidence of cerebellar atrophy. [**B**(**iii**)] shows diffusely elevated T_2_ signal in the entire supratentorial white matter (arrows), suggesting hypomyelination. Size and signal of the basal ganglia and thalami are normal. [**B**(**iv**)] T_1_ signal was hyperintense in the central white matter, hypointense in the parietooccipital white matter and isointense to the cortex in most of the remaining white matter, with some areas of hyperintensity in the subcortical white matter.

Patient 1 was the first child of healthy parents from a rural part of Scandinavia [Fig. 1A (i)]; the parents were not aware of consanguinity. At delivery he was cyanotic with low Apgar scores (Table 1). Birth weight was 4200 g (75th centile) and head circumference 37.5 cm (between 75th–90th centile), based on national growth charts.^18^ Irritability and increased muscle tone with thumb adduction was noted since birth. Jitteriness and increased tendon reflexes were also noted, and cerebral palsy due to perinatal asphyxia was suspected. Converging strabismus was noted at age 13 months. At 13.5 months, his head circumference was 47 cm (50th centile), height was 80 cm (75th centile) and weight 9250 g (between 10th and 25th centile).^18^ At age 5 years, he could speak a few words. He could sit and walk only with support, indicating truncal ataxia. Vertical nystagmus was also noted. From this age regression started. At 7 years, severe dystonia with neurogenic scoliosis developed. At 8 years, epileptic seizures occurred, responding temporarily to treatment with carbamazepine. Myoclonic seizures difficult to treat appeared at 12 years. EEG was normal at the onset of seizures. Later, slow-wave activity without epileptic discharges and eventually bifrontal spike-wave activity occurred.

**Table 1:** Clinical course of the patients with biallelic variants in *POLR1A*

Patients	Patient 1	Patient 2	II-3 (in Kara *et al*.^5^)	II-4 (in Kara *et al*.^5^)
Age (sex)	Died at 16.5 y (male)	2 y 10 m (female)	24 y (male)	Died at 19 y (male)
**Genetic findings**				
Homozygous *POLR1A* variant	T642N (rs750690447)	T642N (rs750690447)	S934L	S934L
CADD Phred	26.6	26.6	25.1	25.1
Total allele frequency gnomAD v.2.1.1	0.00006 (15/249528)	0.00006 (15/249528)	Not reported	Not reported
**Development**				
Perinatal	Apgar scores 2–6 at 1 and 5 min, adducted thumbs, irritability, intra- and extra-cerebral haemorrhages	Normal	NA	NA
Infancy	Hyperreflexia, suspected CP	Hypotonia, delayed smiling	Hyperreflexia	Hyperreflexia, no head control
Childhood and adolescence	Spasticity, dystonia, seizures, neurological regression	Motor delay, little contact, swallowing difficulty, spasticity	Motor delay, spasticity, seizures, neurological regression	Seizures, spasticity, non-ambulatory, no words
**Clinical findings**				
Age	16.5 y	2 y 10 m	11 y	6.5 y
Cognitive functioning	Profound ID	Global delay	Severe ID	Severe ID
Cerebellar dysfunction	Ataxia	Ataxia	Ataxia, head titubation	Ataxia, head titubation
Extrapyramidal signs	Severe dystonia, neurogenic scoliosis	Dystonic patterning	Not reported	Not reported
Growth anomalies	Not present	Brachycephaly, short stature	Low birth weight	Low birth weight, short stature
Ophthalmological findings	Strabismus, cataract, vertical nystagmus	Nystagmus	Optical atrophy, gaze palsy	Optical atrophy, gaze palsy
**Brain imaging**				
Cerebellum and brainstem	Cerebellar and brainstem atrophy, prominent cisterns	Cerebellar atrophy, prominent cisterns	Cerebellar atrophy	Cerebellar atrophy, enlarged fourth ventricle
Corpus callosum	Thinning	Thinning (subtle)	Thinning	Thinning
Basal ganglia	Small volume/T_2_ hypointensity of globi pallidi	Normal	Normal	Normal
Cerebral cortex	Enlarged subarachnoid spaces	Normal	Enlarged subarachnoid spaces	Enlarged subarachnoid spaces
Lateral ventricles	Enlarged (moderate)	Normal	Enlarged	Enlarged
White matter abnormality	Diffuse signal changes, hypomyelinating leukodystrophy	Diffuse signal changes, hypomyelinating leukodystrophy	Diffuse signal changes, demyelinating leukodystrophy	Diffuse signal changes, demyelinating leukodystrophy

CADD = Combined annotation dependent depletion; CP = cerebral palsy; ID = intellectual disability; m = months; NA = not available; y = years.

Bilateral cataracts were detected at 11 years and surgically corrected. There was no suspicion of hearing deficit. At 15 years, he suffered a prolonged apnoea, but recovered. At 16.5 years, another episode of central apnoea occurred secondary to cardiac arrest. After 5 to 10 min of resuscitation, his cardiac rhythm converted from asystole to normal. He was intubated for 8 days and died 1 day after being extubated. He never regained consciousness after the cardiac arrest.

Brain imaging proceeded as follows: a CT at age 3 weeks showed wide Sylvian fissures and basal cisterns, blood in the interhemispheric fissure and low-density signal changes in periventricular white matter. At 13 months, CT showed generalized brain atrophy, particularly of the cerebellum. MRI at 6.5 years detected generalized T_2_ hyperintense signal in the entire cerebral white matter suggesting hypomyelination, accompanied by thinning of the corpus callosum, a small cerebellum and a large cisterna magna. MRI at 10.5 years indicated that nearly all cerebral white matter had disappeared but some myelin was still present in the cerebellum. The cerebral cortical sulci were deep. The last MRI, at 16.5 years, 3 days after the episode of cardiac arrest, showed increased atrophy of the corpus callosum, cerebellum and brainstem and medulla oblongata, and prominent cisterns [Fig. 1A(ii)]. Axial T_2_ showed small volume of the basal ganglia and thalami and T_2_ hypointensity of the globi pallidi [Fig. 1A(iii)], mimicking neurodegeneration with brain iron accumulation, not detected in the previous examinations. There was cerebral cortical atrophy, loss of almost all white matter, and white matter signal changes with diffuse hyperintense T_2_ signal and hypointense T_1_ signal, indicating hypomyelinating leukodystrophy [Fig. 1A(iv)].

Patient 2 was the second child of unrelated parents of European origin, without known ancestors from Scandinavia [Fig. 1B(i)], born at term after an uneventful pregnancy, with normal birth weight, length and head circumference. Her development was delayed from the beginning; she smiled late and her motor development was severely delayed. She had problems feeding from a bottle. At the age of 2 years, she was not able to sit without support, but was able to roll over. Visual contact and interaction had improved. She could hold her bottle and tried to transfer objects from one hand to the other. At age 2 years and 10 months, she reacted to her name and indicated her favourite short films, but made visual contact only for short periods. Hearing was not formally tested, but clinically normal. Her food intake was acceptable, but parents reported long feeding times and choking. She could roll over with difficulties, and was still not able to sit without support due to truncal ataxia. She had pronounced axial hypotonia and poor head control. She had pendular nystagmus and difficulties with horizontal pursuit. Her hands were often in fists, and she exhibited a dystonic pattern when trying to reach for objects. There was no intention tremor. In vertical suspension, there was scissoring of her legs indicating spasticity and her tendon reflexes were increased with extensor plantar responses. The tone in the arms was normal, but the tone in the legs showed mild velocity-dependent increase on passive movement. Muscle tone increased with agitation in arms and legs. There was no change at age 3.5 years. She did not have seizures. Head circumference was normal, but her length was at −3.3 SD with weight at −2.9 SD. Treatment for dystonia with l-DOPA led to mild improvement of head control and alertness. Due to poor growth and feeding difficulties, a gastrostomy was performed recently.

Brain imaging proceeded as follows: an MRI scan at age 3 years showed diffuse T_2_ hyperintense signal of the supratentorial white matter suggesting hypomyelination [Fig. 1B(iii)]. T_1_ signal was hyperintense in the central white matter, hypointense in the parietooccipital white matter and isointense to the cortex in the remaining white matter [Fig. 1B(iv)]. There was thinning of corpus callosum, cerebellar atrophy and an enlarged cisterna magna [Fig. 1B(ii)] and mildly enlarged lateral ventricles [Fig. 1B(iii and iv)].

### Identification of homozygous *POLR1A* NM_015425.3:c.1925C>A p.(T642N) in patients

In 1987, when Patient 1 was 1 year old, a G-banded karyotype at standard resolution was performed and concluded as normal 46,XY.

Exome sequencing of the family trios for both patients led to the identification of the same homozygous variant in *POLR1A* chr2(GRCh37):g.86292530G>T, NM_015425.3:c.1925C>A, p.(T642N) (Fig. 1A and B). The substitution was predicted to be damaging with a combined annotation dependent depletion Phred score of 26.6. Sanger sequencing confirmed the variant to be homozygous in the patients and heterozygous in parents in both families. In the Genome Aggregation database v.2.1.1 (gnomad.broadinstitute.org), *POLR1A* T642N (rs750690447) was reported in heterozygosity with total allele frequency of 0.00006 (15 out of 249528 alleles). All 15 alleles were identified in the European non-Finnish population, which has allele frequency of 0.0001324 (15 out of 113256 alleles), and 10 out of the 15 alleles were identified in the Swedish population, making rs750690447 a variant with a likely Swedish founder.

### *In silico* analysis of the effect of the variant on the protein structure

**Figure 2:**
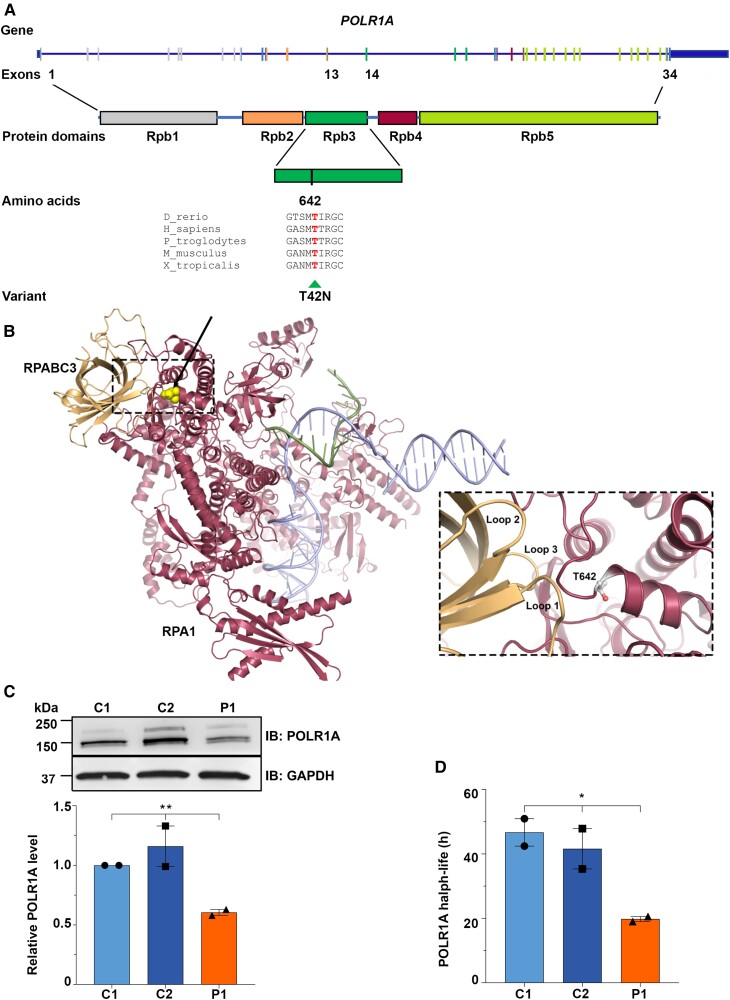
**Schematic representation of *POLR1A* and the encoded protein, *in silico* analysis of POLR1A T642N and expression of mutant *POLR1A***. (**A**) Schematic drawing of *POLR1A* (NM_015425.6) and the encoded protein with domains (NP_056240.2) showing the position of the variant T642N in exon 14 detected in homozygosity in patients. Phylogenetic analyses showed high evolutionary conservation of T642, suggesting that this residue is essential for the function of the protein. *POLR1A* is shown as a blue line and the vertical bars depict the exons. Exons have the same colours as the encoded functional domains. (**B**) POLR1A model (raspberry red) in association with nascent RNA (green) and DNA (light blue) molecules. On the left, interaction with RPABC3 subunit (orange) is shown. The position of the variant is yellow (indicated by the arrow). The *inset* shows RPABC3 loops 1–3 that are all close to the T642 position in POLR1A. (**C**) Representative immunoblot (*top*) and relative quantification of POLR1A protein (*bottom*) in cells from Patient 1 (P1) and controls (C1 and C2). GAPDH was used as loading control. (**D**) Histogram showing POLR1A half-life as measured by CHX time course. (**C** and **D**) *P ≤ 0.05, **P ≤ 0.01 (two-tailed Student's *t*-test).

We modelled the POLR1A T642N variant on a human RNA polymerase I homology model built from the cryogenic-electron microscopy structure of human RNA polymerase II (PDB ID 1FLM).^8^ Amino acid sequence alignment showed that T642 is highly conserved among vertebrates (Fig. 2A). The altered residue is located in the Rpb1 domain 3 (Rpb3) (Fig. 2A), which represents the pore domain of RNA polymerase I. The 3'-end of the newly transcribed RNA is positioned close to this domain.^19,20^ The pore constitutes a channel through which nucleotides enter the active site and/or where the 3'-end of the RNA may be extruded during back-tracking.^19,20^ T642 is located in an alpha-helix involved in binding to several loops in RPABC3 (also called POLR2H or hRPB8), and the substitution for a small amino acid with a slightly larger one (Fig. 2B) possibly reduces the rigidity or stability of the alpha-helix or the adjacent loops, important for protein–protein binding.

### POLR1A T642N exhibited reduced protein stability

To assess the effect of T642N variant on POLR1A function, we used skin fibroblasts from Patient 1 and two controls. Fibroblasts from all individuals displayed normal cell growth (Supplementary Fig. 1A). Western assays revealed a decreased POLR1A T642N protein level in Patient 1 compared to controls (Fig. 2C). To determine whether the lower protein level was due to reduced protein stability, we measured POLR1A half-life using CHX, a protein translation inhibitor. After CHX treatment, the POLR1A level decreased gradually over time, with half-life of 19.80 h for Patient 1, compared to 46.68 and 41.60 h for Control 1 and Control 2, respectively, while no difference was observed for GAPDH levels (Fig. 2D and Supplementary Fig. 1B). Hence, T642N substitution negatively affected the stability of the POLR1A protein.

### POLR1A T642N impaired nucleolar homeostasis

**Figure 3:**
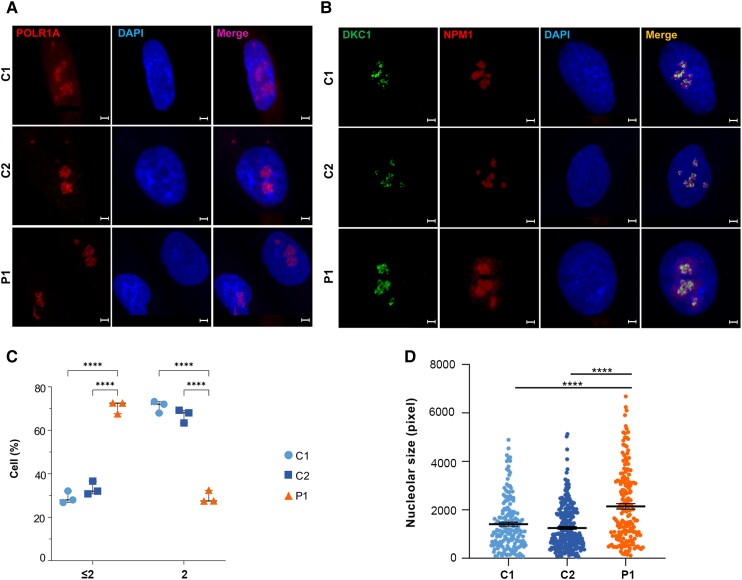
**Immunofluorescence studies on fibroblasts from Patient 1 showed that POLR1A T642N maintained its nucleolar localization but presented defective nucleolar homeostasis**. (**A**) Staining of POLR1A (in red, *left column*) and of the nucleus (in blue, *middle column*) shows that POLR1A localized to the nucleolus in fibroblasts from Patient 1 (P1) and controls (C1 and C2). Scale bars = 5 µm. (**B**) Identification of the nucleoli using the nucleolar markers DKC1 (green, *first column from left*) and NPM1 (red, *second column from left*). Scale bars = 5 µm. (**C**) Quantification of the number of nucleoli (≤2 or >2) per fibroblast. Fibroblasts from Patient 1 showed an increased fraction of cells with ≤2 nucleoli as well as a lower fraction of cells with >2 compared to controls. Bars indicate mean ± SD. *P ≤ 0.05 (two-tailed Student's *t*-test). (**D**) Total nucleolar size determined by boundaries of NPM1 immunostaining. Bars indicate mean ± SEM for *n* > 30 cells per cell type. ****P ≤ 0.0001 (two-tailed Student's *t*-test).

POLR1A localizes to the nucleolus, the site of ribosome biogenesis.^21^ The T642N variant did not disrupt intracellular targeting, as endogenous POLR1A was detected in the nucleolus in fibroblasts from Patient 1 as well as controls (Fig. 3A). However, using the nucleolar markers DKC1 and NPM1 (Fig. 3B), we observed that cells from Patient 1 presented an increased fraction of cells with ≤2 nucleoli per cell (Fig. 3C) and enlarged nucleoli (Fig. 3D), suggesting that T642N impaired nucleolar homeostasis.

### POLR1A T642N exhibited rRNA processing defects

**Figure 4:**
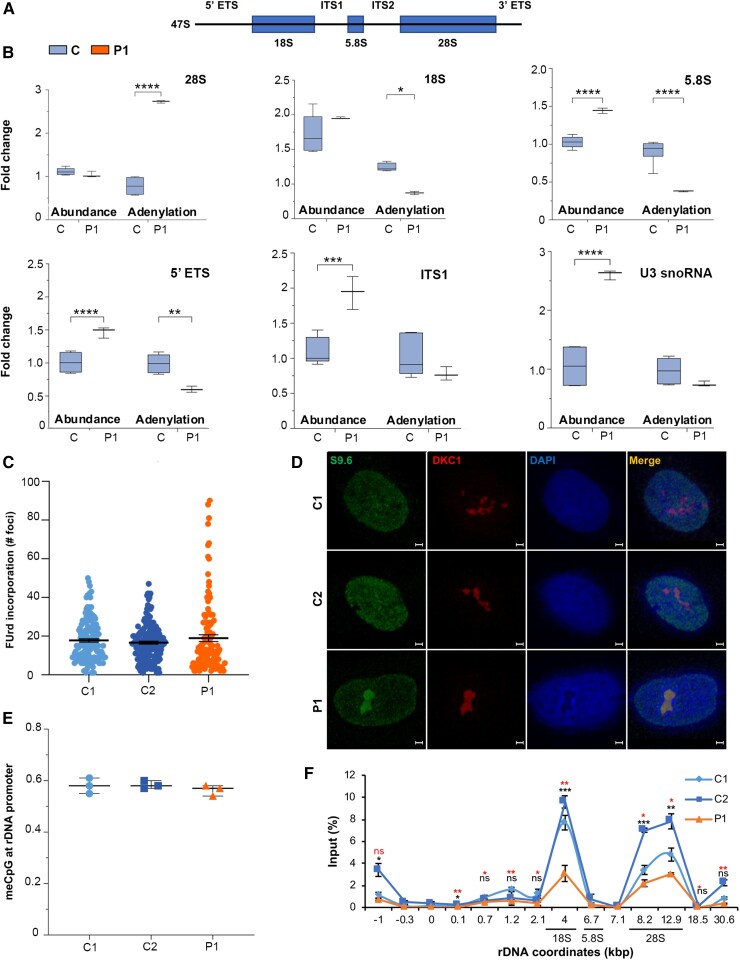
**rRNA processing anomalies in cells expressing POLR1A T642N.** (**A**) Schematic representation of 47S pre-rRNA primary transcript. (**B**) Quantification of immature and mature rRNA (abundance and polyadenylation) products and U3 snoRNA levels measured by qPCR. cDNA was synthesized with random or oligo(dT) primers. Relative abundance was measured using cDNA synthesized with random primers while adenylation frequency was estimated as the ratio of oligo(dT)-primed cDNA to random-primed cDNA. (**C**) Nucleolar transcription as measured by quantification of FUrd incorporation in the newly synthesized rRNA molecules. Box plot showing FUrd incorporation measured as number of FUrd foci per cell in Patient 1 (P1) compared to controls (C1 and C2). Data are shown as the number of foci per cell. (**D**) Immunofluorescence images showing accumulation of RNA:DNA hybrids (S9.6, in green, *first column from left*) in the nucleoli of Patient 1. DKC1 (in red, *second column from left*) was used as nucleolar marker. Scale bars = 5 µm. (**E**) Methyl CpG analyses at −59 CpG island at rDNA promoter. (**F**) RNA polymerase I I occupancy analysis on rDNA as measured by ChIP. *Lower row* of *P*-value (asterisks in red) comparison of C1 to P1; *upper row* of *P*-value (asterisks in black) comparison of C2 to P1; ns = not significant. (**B** and **F**) *P ≤ 0.05, **P ≤ 0.01, ***P ≤ 0.001, ****P ≤ 0.0001 (two-tailed Student's *t*-test).

POLR1A is the largest subunit of the RNA polymerase I complex and constitutes its catalytic subunit. rRNA biogenesis starts in the nucleolus where RNA polymerase I generates a long primary transcript (47S) cleaved by endo- and exonucleases to produce the mature rRNA molecules 18S, 5.8S and 28S (Fig. 4A).^21^ On the primary transcript, those rRNAs are separated by the internal transcribed spacer 1 (ITS1) and 2 (ITS2) and flanked by the 5′- and 3′-external transcribed spacers [5′- external transcribed spacers (ETS) and 3′-ETS] (Fig. 4A). Maturation and post-transcriptional modification of rRNAs involve small nucleolar RNAs (snoRNAs), which are non-coding RNAs that accumulate in the nucleoli.^22^ As availability of the POLR1A subunit is the rate-limiting step in ribosome biogenesis and the level of POLR1A T642N was reduced compared to the controls, we investigated whether the T642N variant disturbed rRNA synthesis. We measured the abundance of both immature rRNAs (with retained 5′-ETS and ITS1) and mature rRNAs (28S, 18S and 5.8S), as well as U3 snoRNA (required for the pre-cleavage of 18S rRNA)^23^ and spliceosomal small nuclear RNAs (snRNAs) (Fig. 4B). In cells from Patient 1, we measured increased levels of immature rRNAs (containing 5′ETS and ITS1 sites), and mature rRNA 5.8S, as well as U3 snoRNA (Fig. 4B). These results indicate dysregulation of rRNA processing.

Degradation of rRNA and small non-coding RNAs occurs mainly via the nuclear exosome pathway, initiated by a poly-A tail addition to the RNA.^24^ RNA degradation of both immature rRNAs, and mature 18S and 5.8S rRNAs was reduced in cells from Patient 1 (Fig. 4B). On the other hand, we measured increased level of polyadenylated 28S rRNA, consistent with its increased degradation.^25–30^ Polyadenylation of U3 snoRNA was not significantly different between Patient 1 and controls. Taken together, in cells from Patient 1, we documented affected processing and degradation of rRNA species.

### POLR1A T642N induced RNA:DNA hybrids

Altered RNA polymerase I activity might cause changes in rRNA abundance. We therefore compared ongoing rDNA transcription in fibroblasts from Patient 1 to those from two controls by FUrd incorporation using *in situ* run-on assay.^11,12^ We observed comparable FUrd incorporation levels at nucleolar sites in Patient 1 and controls (Fig. 4C and Supplementary Fig. 2), indicating that POLR1A T642N did not reduce RNA polymerase I activity. In contrast, we found some cells from Patient 1 with a very high number of foci compared to control fibroblasts, indicating aberrant RNA polymerase I function (Fig. 4C).

Abnormal RNA polymerase I function may cause transcriptional stress and increase the formation or persistence of RNA:DNA hybrids that form during transcription, whose resolution is mediated by the exosome and by the TRAMP-dependent degradation.^31–33^ We compared the persistence of RNA:DNA hybrids in Patient 1 to that in control cells via immunostaining with the S9.6 antibody. Cells from Patient 1 showed an accumulation of DNA:RNA hybrids in nucleoli compared to controls (Fig. 4D), suggesting impaired rDNA transcription and transcriptional stress.

To exclude that the impaired rDNA transcription observed in cells from Patient 1 resulted from hypomethylation of the rDNA promoter, we assessed the methylation level at the promoter. Enzymatic digestion with SmaI followed by real-time PCR was used to quantitate methylation of a CpG site at position −59 within the rDNA promoter. No difference in methylation at this site was observed between cells from Patient 1 and the controls (Fig. 4E).

We next measured RNA polymerase I occupancy throughout the rDNA locus (Fig. 4F). ChIP was performed with POLR1A-coated beads. Real-time amplification of immunopurified DNA was analysed using primers that span the rDNA unit (from −1 kb to +30.6 kb). We measured reduced RNA polymerase I occupancy in the central and the 3′-end regions (approximately +4 kb and +12.9 kb from the transcription starting site), in cells from Patient 1 compared to controls (Fig. 4F). These results indicate that POLR1A T642N lowered the ability of RNA polymerase I to engage in transcription, in line with a loss of function behaviour. Taken together, these results indicate that T642N variant leads to transcriptional stress.

### POLR1A T642N affected protein synthesis and degradation processes

**Figure 5:**
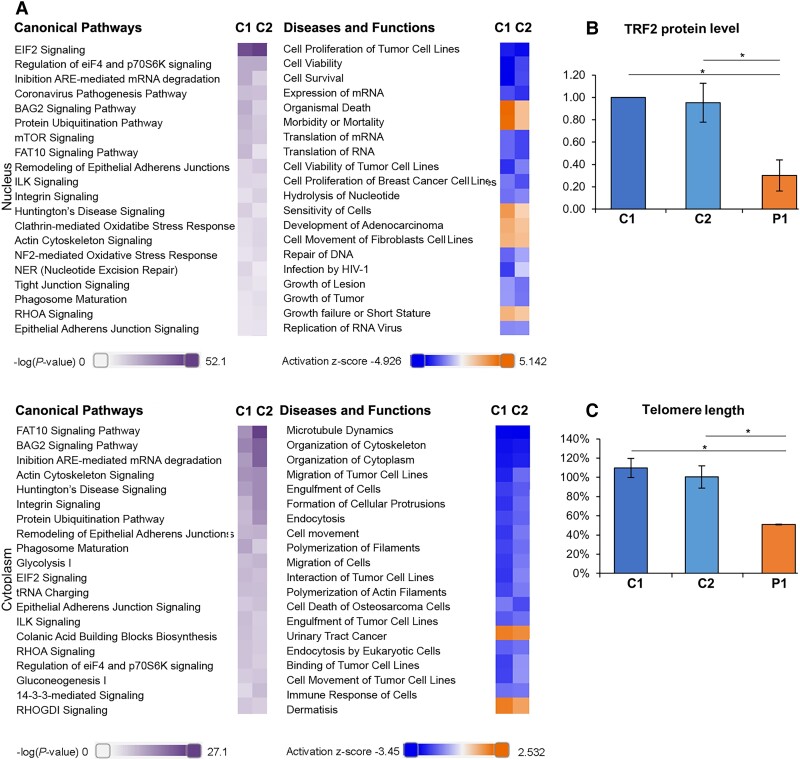
**POLR1A T642N showed increased ER stress and reduced telomere homeostasis.** (**A**) IPA analysis of the proteomics data from fibroblasts of Patient 1 and controls showing the top 20 Canonical pathways (*left*) and Diseases and Functions categories (*right*) dysregulated in Patient 1 versus controls (C1 and C2) in the nuclear proteome (Nucleus) and in the cytoplasmic proteome (Cytoplasm). Lilla scale bar = −log(*P*-value); orange-blue scale bar = Activation *z*-score. For example: orange indicates that a Diseases and Functions category is more active in patient compared to controls. See also Supplementary Table 4. (**B**) Quantification of TRF2 expression levels as measured by western assay in Patient 1 and control cells. (**C**) Quantification of telomere length as measured by qPCR. (**B** and **C**) Mean ± SD, *P ≤ 0.05 (two-tailed Student's *t*-test).

Transcriptional stress can affect protein expression. Therefore, we performed label-free quantitative proteomics analyses to test whether POLR1A T642N affected global protein expression. We analysed both nuclear and cytoplasmic extracts obtained from fibroblasts of Patient 1 and controls and identified major changes (differentially expressed proteins) (Supplementary Tables 2 and 3). We observed decreased POLR1A protein level in the nuclear fraction (Supplementary Table 2), which is in line with the western blot results (Fig. 2D). IPA analyses of the proteomics data indicated dysregulation in the patient cells of canonical pathways related to protein synthesis (EIF2 signalling, regulation of eIF4 and p70S6K signalling, expression and translation of mRNA), protein degradation (ubiquitination, phagosome maturation and mTOR pathway). Dysregulation of pathways related to cellular stress responses (BAG2 signalling, inhibition of ARE-mediated mRNA degradation, FAT10 signalling, NRF2-mediated oxidative stress response) and DNA repair (Fig. 5A and Supplementary Table 4) were also observed. Interestingly, 30 of the dysregulated nuclear proteins (up- or downregulated) in Patient 1 (Supplementary Table 5) were found in a genome-wide siRNA screen to identify regulators of the number of nucleoli.^34^ Deregulation of these proteins is therefore in line with the reduced number of nucleoli in fibroblasts from Patient 1 (Fig. 3C). This suggests that not just depletion, but any change in the abundance of these proteins disturbs nucleoli homeostasis.

### Cells with POLR1A T642N had short telomeres

Analysis of the nuclear proteome in Patient 1 revealed decreased DNA repair (Fig. 5A and Supplementary Table 4) and decreased levels of the telomeric repeat binding factor 2 (TERF2) (Supplementary Table 5). We therefore assessed DNA damage response and telomere homeostasis in cells from Patient 1 and controls after irradiation, following formation and co-localization of γH2Ax foci at DNA damage sites with p53-binding protein 1 (53BP1). γH2AX is the Ser139 phosphorylated version of the histone H2AX in response to double strand breakage,^35^ and 53BP1 is a major regulator of the DNA damage response.^36^ On irradiation, no difference was detected between cells from patient and controls (Supplementary Fig. 3A). These finding were corroborated by the ability of Patient 1’s fibroblasts to promptly induce phosphorylation of p53 at Ser15 after DNA damage, even though total p53 level was lower in those cells (Supplementary Fig. 3B and C). Telomere homeostasis is safeguarded by the Shelterin complex,^37^ which supports chromosomal stability and limits DNA damage response signalling from damaged telomeres.^37–39^ One of its main components, TRF2, showed reduced levels in the nuclear protein fraction of Patient 1’s cells, in line with the results of western assay (Fig. 5B and Supplementary Fig. 3D). Consistent with the decreased level of TRF2, the telomere length was found strongly reduced in cells from Patient 1 compared to the controls (Fig. 5C).

### Cells with POLR1A T642N exhibited abnormal ER stress response

**Figure 6:**
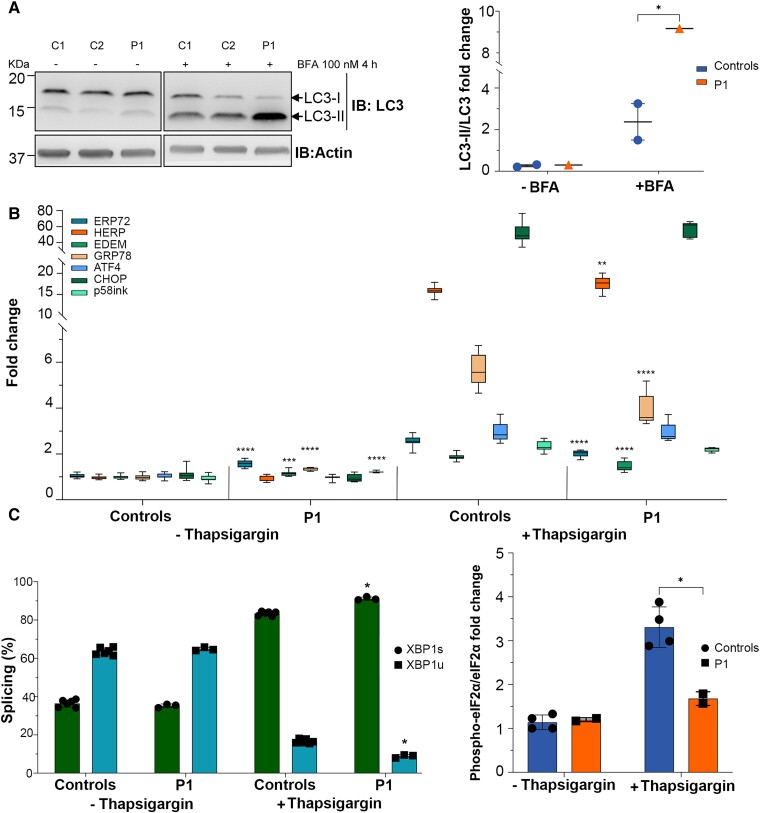
**POLR1A T642N led to increased autophagy and UPR stress.** (**A**) Representative immunoblot for LC3-I and LC3-II (*left*) and quantification of the autophagic flux as ratio LC3-II/LC3-I (*right*). β-Actin was used as loading control. (**B**) Fold changes depicting the activation of UPR pathway. Thapsigargin was used as positive control for the UPR activation.^71^ ERP72 (PDIA4), HERP (HERPUD1), EDEM (EDEM1), GRP78 (HSPA5), ATF4 (ATF4), CHOP (DDIT3) and p58ink (DNAJC3) levels are shown (the HGNC name of each protein is shown within parentheses). (**C**) Histogram showing the splicing ratio of XBP1 mRNA in P1 and control fibroblasts after treatment with thapsigargin. XBP1s = spliced; XBP1u = unspliced. (**D**) Quantification of phospho-eiF2α/eiF2α ratio as measured by western blotting in P1 and control cells treated with thapsigargin. (**B**–**D**) Mean ± SD, *P ≤ 0.05, **P ≤ 0.01, ***P ≤ 0.001, ****P ≤ 0.0001 (two-tailed Student's *t*-test, patient versus controls).

The proteomic data analyses in cells from Patient 1 showed impairment of the protein synthesis and degradation and cellular stress responses (Fig. 5A and Supplementary Table 4). We confirmed abnormal autophagic flux in Patient 1 by inhibiting the autophagosome-lysosome fusion using BFA (Fig. 6A).^40^ The BFA treatment resulted in increased stabilization of the autophagy marker LC3-II in cells from Patient 1, indicating an accumulation of autophagosomes, caused either by upregulation of phagosome maturation or by blockage of autophagic degradation (Fig. 6A). ER stress leads to the activation of the unfolded protein response (UPR), as an attempt to restore protein homeostasis and ensures cellular survival.^41^ The UPR activity relies on the activation of different effectors, mediated by three ER transmembrane proteins: Inositol requiring enzyme 1α/β (IRE1), Activating transcription factor 6α/β (ATF6) and PKR-like ER kinase (PERK).^41^ Activation of these factors leads to transcriptional activation of genes involved in the attenuation of protein synthesis, augmenting cellular capacity for protein folding, transport and degradation via autophagy and the endoplasmic reticulum-associated degradation pathway.^41^ We investigated the UPR activation status in cells before and after thapsigargin-induced ER stress caused by specific inhibition of the sarcoplasmic/ER Ca^2+^-ATPase.^42,43^ Fibroblasts from Patient 1 but not from the controls exhibited basal activation of several factors of the UPR, ERP72, EDEM, GRP78, P58ink, XBP1 and eiF2a, indicating a constitutive underlying ER stress in these cells (Fig. 6B–D and Supplementary Fig. 3E–F). The response to the ER stress inducer thapsigargin was enhanced in cells from Patient 1 compared to controls (Fig. 6B–D). Interestingly, CHOP and ATF4, both involved in apoptotic responses, showed no difference between controls and Patient 1 cells (Fig. 6B).

Taken together, we document that POLR1A T642N is a pathogenic variant, and our *in vitro* data indicate that this variant causes defects in rRNA processing and degradation, leading to disturbances in protein homeostasis and ER stress responses.

## Discussion

We describe the homozygous POLR1A T642N missense variant in two unrelated patients. Both presented with early-onset global developmental delay and white matter abnormalities suggestive of hypomyelinating leukodystrophy.^44^ In the older patient, loss of neural tissue, seen on MRI as thinning of the corpus callosum and atrophy of brainstem and cerebellum, resulted in spinocerebellar dysfunction and spasticity. There were also extrapyramidal signs with dystonia in both patients. The two brothers were previously reported with homozygous *POLR1A* c.2801C>T; p.(Ser934Leu),^5^ and manifesting clinical similarities to the patients presented before (Table 1). Our findings verify that POLR1A defects lead to neurodegeneration.

POLR1A dysfunction is rare, and both dominant and recessive inheritance are described. On the basis of the few patients reported, the dominant and recessive diseases result in markedly different phenotypes: mandibulofacial dysostosis or leukodystrophy, respectively.^3–5^ Pathogenic variants also in other subunits of RNA polymerase I as well as of RNA polymerase III cause diseases; the complexity of the associated clinical manifestations is expanding as more morbid genes are identified (Supplementary Table 6). Main clinical presentations of POLR-related defects are: hypomyelinating leukodystrophy, skeletal anomalies, which include dysostoses and dental anomalies (hypodontia), endocrinopathy (hypogonadotropic hypogonadism),^45–48^ and premature ageing.^3,5,49–58^ The molecular pathogenesis in disorders caused by RNA polymerase I and III dysfunction is not fully elucidated. Two pathophysiological hypotheses for RNA polymerase III-related hypomyelinating leukodystrophies have been proposed.^59^ In the first hypothesis, the hypofunctional RNA polymerase III produces reduced levels of tRNA and small non-coding RNAs resulting in globally impaired translation. Oligodendrocytes might be susceptible to decreased translation capacity during myelin formation, when the demand for newly synthesized proteins is high. In the second hypothesis hypofunctional RNA polymerase III produces reduced levels of specific transcripts, affecting RNA processing and/or translation, thereby impairing specific cellular functions. The two hypotheses are not mutually exclusive and both are compatible with our data, which indicated disturbed protein homeostasis in cells expressing POLR1A T642N.

The disease mechanism of heterozygous *POLR1A* variants was unravelled by studying the effect of the loss of polr1a in zebrafish embryos.^3^ Interestingly, these embryos displayed cerebral hypoplasia and craniofacial skeletal anomalies,^3^ paralleling the human disease phenotypes. At the cellular level, loss of polr1a decreased the level of immature rRNA, leading to ribosome biogenesis deficiency, nucleolar stress and increased p53 levels. This resulted in increased p53-dependent neuroepithelial apoptosis, diminished neural crest cell proliferation and cranioskeletal anomalies.^3^ However, the mechanism by which mutated POLR1A causes a human leukodystrophy has not yet been addressed. This motivated us to perform *in silico* modelling of the T642N variant and a series of *in vitro* experiments using fibroblasts from Patient 1.

Using *in silico* modelling, POLR1A E593Q, which causes dominant mandibulofacial dysostosis, was recently reported to generate a dominant negative effect.^60^ Both the E593Q mutation and the T642N reported here are predicted not to be tolerated and to affect protein function. E593Q affects a highly conserved residue localized in the active site where E593 seems to bind between RNA and DNA in the complex.^61^ In our *in silico* modelling, we showed that T642 localizes to an α-helix. The T642N substitution may reduce the rigidity and/or stability of this α-helix and the adjacent loop that binds RPABC3. This might result in a less stable POLR1A protein, in line with our results in fibroblasts of Patient 1 (Fig. 2). Consistently, our ChIP data show that the T642N mutant may be less stably bound to rDNA or dissociate faster (Fig. 4F). This may decrease the interaction between POLR1A and RPABC3. RPABC3, a component of all three eukaryotic RNA polymerases, has a non-specific single strand (ss) DNA binding site (aa 16–40) and the RPABC3–ssDNA complex has fast association and dissociation kinetics.^62^ Since RPABC3 binds ssDNA, freeing the newly synthesized RNA, it has been suggested that RPABC3 is involved in one exit path of the nascent RNA,^63^ which could be affected in cells expressing POLR1A T642N.

E593 is located close to the magnesium-binding aspartate residues (D592, D590 and D588), and the change in charge caused by the E593Q mutation may affect magnesium coordination geometry and nucleotide addition, which could decrease RNA polymerase I transcription and ultimately generate a dominant negative enzyme.^60^ By contrast, increased incorporation of FUrd in patient fibroblasts showed that the T642N mutation was associated with higher rates of rRNA transcription (Fig. 4C and D), strongly suggesting that the enlarged nucleolar size (Fig. 3) could be caused by a more open chromatin organization. It is conceivable that the reduced levels of POLR1A T642N might lead to high transcription rate as a compensatory response since transcription in the rDNA locus is tightly regulated. The subcellular localizations of the mutated proteins also point to different mechanisms: ectopically overexpressed POLR1A E593Q was shown to result in movement of RNA polymerase I to the nucleolar caps, ^61^ which is a hallmark of transcription inhibition. The inhibitory effect of E593Q seemed to be associated with stable binding of E593Q to rDNA and UBF1 and faster RNA polymerase I movement.^61^ By contrast, we found that POLR1A T642N was localized at its canonical, nucleolar localization, although the number of the nucleoli was decreased and the nucleolar size was increased (Fig. 3). Consistent with this, we observed a tendency towards increased rRNAs levels in cells from Patient 1 (Fig. 4B). Increased RNA polymerase I activity and rRNA levels were previously shown to positively correlate with increased nucleolar size.^64–67^ These changes in cells expressing POLR1A T642N probably lead to nucleolar stress.

We hypothesize that the POLR1A T642N substitution may result in reduced proofreading activity, ultimately affecting the accuracy of the pre-rRNA transcribed. To keep a steady level of mature rRNA, increased transcription may provide a compensatory mechanism, considering also the reduced level of POLR1A protein in the patient cells. Thus, the available evidence supports that the E593Q and T642N variants affect POLR1A through different mechanisms. In the case of T642N, our observation of RNA:DNA hybrids formation in the nucleoli of patient fibroblasts (Fig. 4D) and abnormal rRNA processing and degradation defects (Fig. 4B), are consistent with transcriptional stress. We suggest that abnormal rRNA transcription impairs ribosome biogenesis and therefore protein synthesis. In line with this, proteomics data indicated dysregulation of pathways related to protein synthesis and degradation (Fig. 5A). Abnormal protein synthesis will in turn cause ER stress and activate the UPR.^41^

A constitutive activation of the UPR response at basal conditions could be seen in patient cells (Fig. 6B), in addition to an UPR dysregulation after ER stress induction (Fig. 6B–D), pointing to a possible mechanism where patient cells activated UPR to counteract the anomalies in protein translation due to rRNA processing defects. Cells expressing POLR1A T642N presented normal DNA damage response to insults such as ionizing radiation including the activating phosphorylation of p53 at Ser15 (Supplementary Fig. 3A). However, the total level of p53 was reduced (Supplementary Fig. 3B and C). In line with this, the pro-apoptotic marker CHOP was not elevated in patient cells, and in the cytoplasmic component of the proteome apoptosis was found decreased (Figs 5A and 6B).

There are limitations posed by the use of fibroblasts. Even though the general functions of POLR1A are similar across cell types, the pathological mechanisms may differ between fibroblasts and cells and tissues relevant for the disease under study. However, ∼60% of all known disease associated genes are expressed in fibroblasts, and this biological source has shown diagnostic utility in patient groups with rare diseases, including neurological diseases.^68,69^ In addition, fibroblasts have over many years been proven to be a valuable study material in rare neurological diseases, as recently illustrated.^70^ Our initial studies of the cellular consequences of POLR1A dysfunction provide information that is valuable for further studies.

**Figure 7:**
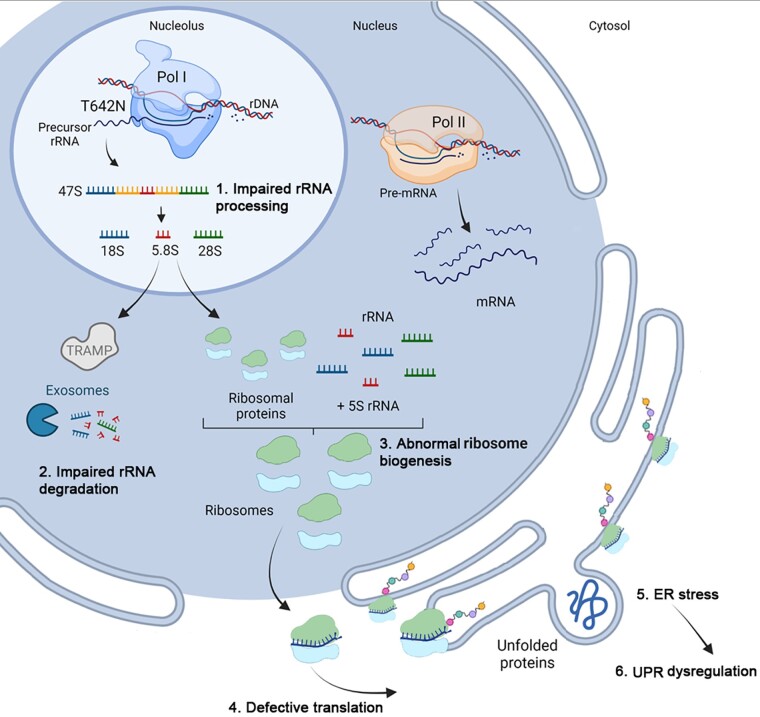
**The cartoon illustrates the suggested cascade of events in cells expressing POLR1A T642N based on the *in vitro* studies**. The steps in the cascade are labelled 1 to 7. 1. RNA polymerase I with the mutated POLR1A subunit produces rRNA precursors that will undergo abnormal processing and 2. impaired rRNA degradation, which was documented by altered levels of mature and immature rRNA species, and their polyadenylated forms, in fibroblasts from Patient 1 compared to controls. 3. Abnormal rRNA mature species will possibly lead to abnormal ribosome biogenesis. 4. This in turn will cause nucleolar stress and defective translation. The nucleolar stress was documented in fibroblasts from Patient 1 by the presence of a reduced number of nucleoli per cell, which also were enlarged. 5. Defective translation was probably the cause of ER stress induction. 6. ER stress dysregulated the UPR pathways. In fact, protein levels of several UPR pathway components were found to be significantly different in cells from Patient 1 compared to the controls, both in resting condition and after thapsigargin-induced ER stress.

In conclusion, our results confirm that biallelic POLR1A variants cause a neurodegenerative disease. Our *in vitro* data indicate a model where the pathogenic POLR1A T642N variant impairs protein homeostasis, from synthesis to degradation (Fig. 7). We documented disturbed rRNA turnover that caused nucleolar stress, which in turn probably impairs protein translation. This causes ER stress with further activation of UPR, in an attempt to restore cellular homeostasis. The suggested pathological mechanism in POLR1A-related leukodystrophy warrants further validation.

## Supplementary Material

awad086_Supplementary_Data
